# BIOLAP: biological versus synthetic mesh in laparo-endoscopic inguinal hernia repair: study protocol for a randomized, multicenter, self-controlled clinical trial

**DOI:** 10.1186/s13063-018-3122-5

**Published:** 2019-01-16

**Authors:** C. S. Seefeldt, J. S. Meyer, J. Knievel, A. Rieger, R. Geißen, R. Lefering, M. M. Heiss

**Affiliations:** 1 Department of Visceral, Vascular and Transplant Surgery/Chair of Surgery of the University of Witten-Herdecke, Krankenhaus Merheim, Kliniken der Stadt Köln, Ostmerheimer Straße. 200, 51109 Köln, Germany; 2 Institut für Forschung in der Operativen Medizin der Universität Witten/Herdecke, Ostmerheimer Straße 200, Haus 38, 51109 Köln, Germany; 3 Chirurgische Klinik, St. Elisabeth Hospital Köln, Former Krankenhaus Merheim, Kliniken der Stadt Köln, Werthmannstr. 1, 50935 Köln, Germany; 4 Zentrum für klinische Studien der Universität Witten/Herdecke, Alfred-Herrhausen-Str. 50, 58448 Witten, Germany

**Keywords:** Inguinal hernia, Hernia inguinalis, Biomesh, Biological mesh, Self-controlled trial, Randomized trial, TAPP, TEP, Blinded trial, Laparo-endoscopic hernia repair

## Abstract

**Background:**

Inguinal hernia repair is one of the most common surgical operations globally; more than 20 million groin herniae are repaired annually worldwide. Recurrence after an inguinal hernia operation is a considerable clinical problem. Another remaining problem after hernia surgery is the occurrence of chronic pain. Up to now, the use of synthetic meshes is the standard procedure, but there is increasing evidence that biological meshes could be advantageous concerning the occurrence of chronic pain due to different postoperative remodeling, without the disadvantages of a life-long implant.

We hypothesize that the use of a biological mesh reduces postoperative pain without being inferior in terms of recurrence rate compared with a synthetic mesh.

**Methods/design:**

The trial compares possible the advantages of biological matrices to synthetic meshes in laparo-endoscopic inguinal hernia repair. Four hundred and ninety-six patients with primary bilateral inguinal herniae in 20 German hernia centers will be enrolled. Biological mesh is used for one of the bilateral herniae, the other side will be operated on with a synthetic mesh. Randomization will preset which side is repaired with which material and trial participants will not be informed about the location of each mesh type. The primary endpoints will be intensity of postoperative local pain and the incidence of recurrent hernia after 2 years.

**Discussion:**

There is no reasonably sized trial that assesses the use of biological meshes in laparo-endoscopic inguinal hernia repair.

Our self-controlled trial design allows a direct comparison of the two meshes with very few confounding factors as well as minimizing the exclusion criteria. As we compare CE-certified medical devices in their designated indication the medical risk is not different compared to routine clinical care. Due to the common nature of bilateral inguinal hernia, a high recruitment rate is achievable. Because guidelines for hernia repair have stressed the need for reliable data on the already frequent use of biological meshes, we can expect our trial to have a direct implication on hernia-repair standards.

**Trial registration:**

German Clinical Trials Register, ID: DRKS00010178. Registered on 16.June.2016. BIOLAP underwent full external peer review as part of the funding process with the German Research Foundation.

**Electronic supplementary material:**

The online version of this article (10.1186/s13063-018-3122-5) contains supplementary material, which is available to authorized users.

## Background

The lifetime risk of developing an inguinal hernia is 3% for women and 27% for men [1]. Accordingly, inguinal hernia repair is one of the most common surgical operations globally, more than 20 million groin herniae are repaired annually worldwide [2, 3]. The 2010 Global Burden of Disease Trial found that 11 disability-adjusted life years (DALYs) per 100,000 population per year were attributable to groin hernia [4]. Up to 30% of patients who present for surgical treatment suffer from bilateral inguinal herniae, and the true incidence of bilateral inguinal herniae is unknown [5, 6].

If surgical treatment of groin herniae is indicated in adults, options are primary open repair, open repair with mesh, or laparo-endoscopic repair with mesh [1, 6, 7]. Simultaneous laparo-endoscopic repair of symptomatic bilateral herniae is considered safe and effective with postoperative pain and length of reconvalescence comparable to unilateral operation [8–11]. Various studies have found mesh implantation to be superior to sutured repairs in terms of recurrence and chronic inguinal pain, the two main challenges remaining in surgical hernia repair [3, 10, 12, 13]. Up to now, the use of synthetic meshes is the standard procedure [6], but there is increasing evidence that biological meshes could be advantageous concerning the occurrence of chronic pain due to different postoperative remodelling without the disadvantages of a life-long implant [14].

When searching the PudMed database there are very few studies comparing biological to synthetic mesh in open inguinal hernia surgery. Bochicchio et al. report in their randomized, double-blinded trial, using Lichtenstein’s repair, a favourable outcome for biological mesh: The 1-year follow-up showed a lower, yet not statistically significant, rate of persistent incisional pain and neuralgia. However, the slightly higher rate of recurrence of the biological mesh may have been correlated with the surgeon’s experience [14]. The only trial examining the use of biological mesh in transabdominal preperitoneal patch plasty (TAPP) hernioplasty is a retrospective case series of 11, reporting the feasibility with good results [15]. A 2015 literature review by Köckerling et al. reported an equivalent recurrence rate, but stressed the small sample size and the need for further randomized controlled studies in order to justify the higher cost of biological meshes [16]. A systematic literature review and meta-analysis of five randomized controlled trials (RCTs) by Fang et al. reported no difference in chronic groin pain and recurrence rate, but shows a higher incidence of seroma for biological meshes [17]. There are currently no self-controlled, randomized, multicenter studies like the one that we are conducting now.

Because biological meshes are already frequently used for hernia repair, particularly in cases with risk for implant infection [16], and there is evidence for possible advantages of biological mesh, a reasonable sample-sized randomized trial is desirable. If the trial results prove non-inferiority in recurrence rate and maybe even superiority in the occurrence of postoperative pain, biological meshes will have to be considered as standard implants in inguinal hernia repair.

## Methods/design

### Aim of the trial

The aim of this trial is to research the possible advantages of biological matrices versus synthetic meshes in laparo-endoscopic inguinal hernia repair. We hypothesize that by using a biological matrix for laparo-endoscopic inguinal hernia repair, the occurrence of postoperative pain is significantly reduced (superiority) without it causing a higher recurrence rate (non-inferiority).

### Primary and secondary outcomes

Primary outcomes represent the two most challenging aspects in hernia surgery: Local pain at 6 months after surgery and recurrence within 2 years. Pain measurements will be derived from all trial visits on the basis of a visual analog scale (VAS) and will be documented separately for each side that is operated on. Pain will further be restricted to the inguinal region and is thus not contaminated with other sources of pain, like headache or back pain. The presence of a recurrent hernia requires the suspicion from clinical investigation plus a diagnostic confirmation by ultrasound, computed tomography scan (CT scan) or magnetic resonance imaging scan (MRI scan). All information regarding pain and recurrence will be documented as left/right, and which type of mesh was used will be kept sealed until the end of the trial.

Secondary outcome measures are local pain, haematoma and seroma incidence, complications due to surgery (e.g., infection, mesh dislocation) and patient satisfaction at every follow-up point. A complication is noted when an intervention is necessary or the hospital stay is significantly prolonged.

Due to the self-controlled design of the trial, a perfect setting for patient satisfaction evaluation is given. The following aspects will be assessed using a questionnaire: foreign-body sensation, overall satisfaction and somatosensory alterations. For patient satisfaction we ask whether the patient is, for whatever reason, more satisfied with one side than the other. Patients will be offered three options: right side better than left, left side better than right, or equally satisfied.

### Design/setting/participants

The BIOLAP trial is designed as a German national multicenter, randomized, self-controlled clinical trial. The central element of our trial design is its self-controlled feature: Only patients with bilateral herniae will be included. Each patient will receive a biological mesh on one side, left or right. The other side will be repaired with a synthetic mesh. Randomization will preset which side is repaired with which material and trial participants will not be informed about the location of each mesh type. As this trial design makes each patient their own control it allows for an ideal comparison of biological and synthetic meshes without confounding factors [18].

This multicenter trial will be conducted in at least 15–20 hospital departments all over Germany with a special focus on hernia surgery, preferably certified by the German Hernia Society to ensure surgical quality. Four hundred and ninety-six adult patients (age ≥ 18 years) with primary, bilateral herniae suitable for laparo-endoscopic hernia repair will be included. Due to the self-controlled design, the main exclusion criteria can be limited to recurrent herniae, incarcerated herniae and the presence of acute systemic infection. Trial participants must be fully legally competent and need to provide written informed consent before randomization, inclusion in the trial, or any trial-related procedure. All inclusion and exclusion criteria are listed in Table 1.

**Table 1:** List of all inclusion and exclusion criteria

Inclusion criteria	Exclusion criteria
✓ Patients with primary bilateral herniae suitable for laparoscopic hernia repair	× Recurrent or incarcerated herniae
✓ Age ≥ 18 years	× Presence of acute systemic infection
✓ Full legal competence	× Expected non-compliance with the requirements of the trial
✓ Patient has given written informed consent	× Severe comorbidity (ASA ≥ 4)
	× Expectancy of life < 12 months
	× Chemotherapy within the last 4 weeks
	× Radiation therapy within the last 2 months
	× Allergy to porcine or bovine antigens
	× Simultaneous participation in other interventional trials
	× Persons who are in a dependency/ employment relationship with the sponsor or one of the clinical investigators
	× Placement in an institution due to legal or governmental order

*ASA* American Society of Anesthesiologists

**Fig. 1:**
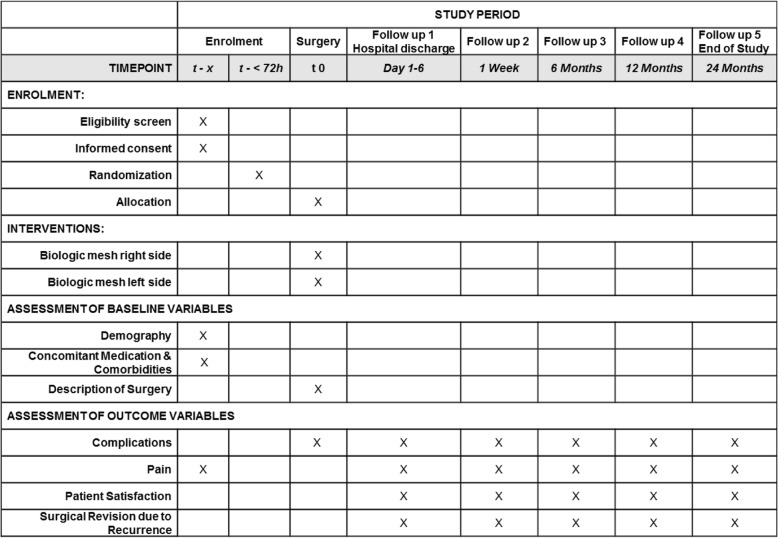
Standard Protocol Items: Recommendations for Interventional Trials (SPIRIT) Figure BIOLAP

The total duration of follow-up per patient will be 2 years. Seven trial visits are planned to assess the outcome measures: in addition to the preoperative assessment and the surgical procedure itself a clinical follow-up visit and assessment of endpoints at discharge, 1 week, 6 months, 1 year and 2 years after the surgery is planned ((see Fig. 1 and Additional file 1), Standard Protocol Items: Recommendations for Interventional Trials (SPIRIT) Figure: BIOLAP). Follow-up visits are planned as personal clinical examinations. If a hernia recurrence is suspected, verification with ultrasound, MRI scan, or CT scan must be performed.

### Intervention

We will compare two types of certified medical devices in their designated indication. The trial sites can use all *Conformité Européenne* (CE)-certified, commercially available meshes that are at least 10 × 15 cm in size. The biological mesh should be a perforated, non-cross-linked, acellular, collagenous matrix. The synthetic mesh should be large-pored, lightweight and made of polypropylene, polyester, or polyvinylidene fluoride. The meshes will be implanted using the standard laparo-endoscopic TAPP or total extraperitoneal patch plasty (TEP) technique according to the “Guidelines for laparoscopic (TAPP) and endoscopic (TEP) treatment of inguinal hernia” by the International Endohernia Society (IEHS) [19]. The same procedure must be used for both sides. Additional glue fixation may be used and is strongly recommended for all size-3 herniae in accordance to the IEHS recommendations. If any kind of fixation is used, it has to be used on both sides. Biological mesh is used in hernia repair for one of the bilateral herniae, and the other side will be operated on with a synthetic mesh. Localization of the meshes will be allocated by randomization. The size and type of the hernia will be documented intraoperatively using the European Hernia Society (EHS) classification of inguinal herniae and will be compared in the final analysis [20]. For size-3 herniae we additionally document the transverse diameter of the hernial orifice in centimeters.

### Randomization

Randomization of the right side (biologic or synthetic mesh) will be performed up to 72 h before surgery via a phone call to the executive assistant of the head of clinical investigation. A block randomization with variable block length, stratified per trial site, is performed. Several random lists of the size *n* = 50 (25 versus 25) were generated by a freely available software, using block sizes of 4, 6, or 8. All sequences were checked for correctness. The lists are randomly assigned to the centers as required.

### Blinding

In this RCT both patients and clinicians are blinded for the location of mesh types. Since there is a common operational access and the technique of implantation is identical on both sides, it is not possible to unblind the treatment allocation by scars or dressings. The operating surgeon, who is, of course, unblinded, should not perform the follow-up visits. Allocation should not be noted in the discharge letter or other documents directly handed to the patient in order to ensure best possible concealment of allocation.

Unblinding is only permissible in case of recurrence to allow an adequate re-operation, the contralateral hernia procedure will inevitably be unblinded as well. With recurrence of one hernia, the endpoint for this side is reached, but not for the patient;.as long as the other side has not developed a recurrence, the patient remains in the trial.

### Statistical analysis and power calculation

The trial will evaluate two different confirmative endpoints in parallel. Therefore, according to Bonferroni, the *p* value has to be adjusted to 0.025% for each endpoint.

The first primary endpoint is the intensity of postoperative local pain; VAS pain measurements (0–10) are known to show large variability. Postoperative values tend to show a standard deviation (SD) of 1.5–2.0 while pain at follow-up tends to be lower (SD 1.0–1.5). A large percentage of patients are expected to have no pain at follow-up. It is expected that more than half of all patients have no pain at all, for both meshes. If pain is reported, then pain intensity is expected to be mostly < 5 points. A difference of 1 point would, therefore, be a large difference. It is thus intended to identify a difference of 0.5 points in this trial. Assuming a SD of 1.5 points, this means a reduction of one third of the SD. The required sample size (paired *t* test; power 90%; alpha 0.025) would be 114 herniae per group, or 114 patients. Since the final analysis will be done using non-parametric rank statistics (Wilcoxon signed-rank test), sample size will be increased by 15% (due to the lesser power of rank tests). Thus, the total number of patients should be at least 131 for the first endpoint.

The second primary endpoint (recurrence rate) is a non-inferiority hypothesis. Recurrence rates are expected to be around 5% after 2 years [21]. The maximum tolerated difference would be + 3%, i.e., an 8% recurrence rate for the biological mesh. If it is assumed that both meshes have the same recurrence rate then 203 cases would be required to exclude + 3% from the 95% confidence interval, i.e., the difference would be 0% (− 3; + 3). If the recurrence rate in the group with biological mesh turns out to be 1% higher than in the control group, then 451 cases would be required to generate a 95% confidence interval of ± 2%, i.e., the difference between the recurrence rates would be + 1% (− 1; + 3). Assuming a 10% rate of loss to follow-up, a total of 496 randomized patients is required. It is finally assumed that an additional 10% of patients must be screened for inclusion, i.e., total number of patients to be screened is *n* = 546.

All endpoints will be analyzed in the intention-to-treat (ITT) population. In case of missing follow-up data, the last-observation-carried-forward (LOCF) method will be used. Due to the self-controlled design, losses to follow-up will be identical for both meshes in this trial.

This trial is one of the rare situations where each patient is their own control (self-controlled design). Therefore, the analysis is similar to matched-pair studies. The present trial has two primary endpoints. In order to evaluate both endpoints independently, the type-1 error rate was set to 2.5% (α = 0.025) for each endpoint according to Bonferroni.

For the first primary endpoint (pain intensity at 6 months) there will be two pain measurements from each patient, from the left and the right side. Due to prior experience, pain data are expected not to show normally distributed data. Furthermore, many patients (> 50%) are expected to have no pain at follow-up. Therefore, a non-parametric approach will be used. The respective statistical test is Wilcoxon’s signed-rank test. The two-sided alpha error for the test will be at *p* = 0.025. Secondary endpoints include pain intensity at various time points post surgery, which will be evaluated in the same way. Furthermore, the prevalence of pain (VAS 2–10) will be evaluated using McNemar’s test for dependent data.

For the second primary endpoint, recurrence, non-inferiority will be evaluated. It is expected, that the recurrence rate of the biological mesh is lower, equal, or slightly higher than the respective recurrence rate of the synthetic mesh. The range of comparability was set at ± 3%. For the observed difference between the two recurrence rates (biological versus synthetic mesh) a 95% confidence interval will be calculated. If this confidence interval does not contain the value of +3%, then the biological mesh will be considered as non-inferior. Since non-inferiority is a one-sided hypothesis, a 95% confidence interval is appropriate here (one side = 2.5%). Recurrence rates over time will be presented using Kaplan-Meier plots.

Patient satisfaction for one side or the other will be transformed into preference for one type of mesh and compared using a sign test.

### Data management

All patient data will be recorded in a paper-based Case Report Form. The data will then be entered in an electronic database twice by two independent individuals, for confirmation.

Only people involved in the trial and authorized by the sponsor will have access to the data via username and password. No patient-identifying information will be stored. Data protection is ensured by pseudonymization of all person-related data according to the European Data Protection Act.

### Monitoring

Monitoring will be performed by the Division of Clinical Research at the Institute for Research in Operative Medicine of the University Witten/Herdecke (UW/H). Monitoring involves initiation and close-out of each participating center as well as periodic on-site visits by monitors for the duration of the trial. These visits are to ensure that investigators are following the protocol, complying with regulatory and Good Clinical Practice (GCP) standards and collecting and reporting quality data. Furthermore, the respective Clinical Research Associate (CRA) motivates the site to recruit the expected number of patients, assists with all trial-related questions and problems, and mediates between other project partners if necessary. Complete monitoring (100%) will be done for the screening and randomization visit (including the inclusion- and exclusion criteria), for the primary endpoints and the safety data (adverse and serious adverse events) for each patient. Overall, ten monitoring visits will be performed at each trial site, including initiation and close-out, seven visits for periodic monitoring and one visit for motivation, and trouble-shooting if necessary.

### Safety

All complications in relation to surgical procedure and the used medical product will be documented during the duration of the trial and have to be reported to the sponsor without delay. These data will be provided for the periodical review of a Data and Safety Monitoring Board. In case of a significant accumulation of complications, the Data and Safety Monitoring Board, sponsor and head of clinical investigation assess whether the trial has to be stopped prematurely.

## Discussion

The trial population was chosen to be broad and, therefore, representative for the patient population. All patients suitable for bilateral laparo-endoscopic hernia repair qualify for BIOLAP, other than those within the exclusion criteria, which are essentially limited to recurrent herniae, as their inclusion would confound the outcome, as well as incapacity to consent. Consequently, results can be expected to be applicable for clinical routine.

The outcome measures consider the major challenges in hernia surgery: pain and recurrence. The primary endpoints are geared towards long-term results, even though an observation period of 2 years might be too short to test for long-term recurrence rate. A further follow-up of recurrence for up to 5 or 10 years is being discussed within the study team as this can be tested with by then unblinded patients.

The limitation to laparo-endoscopic surgery reflects the strong recommendation by the international guidelines for groin hernia management for the treatment of primary bilateral inguinal herniae [19].

The self-controlled trial design allows for best comparability of both methods at the same time: in self-controlled trials the data have a natural dependency structure, which influences the analysis strategy [18]. The direct comparison of the two mesh types at the same time within the same individual prevents confounding factors such as surgeon’s experience, duration of the operation and patient factors like metabolic diseases, chronic constipation, weight, physical activity, wound-healing ability, etc.

Inguinal hernia is a very common pathology; thus, we are confident in achieving the required number of patients within the planned time frame. The required minimum for certification to a participating center by the German Hernia Society is at least 250 laparo-endoscopic surgeries per year. Furthermore, up to 30% of patients suffer from bilateral herniae [5, 6]. Therefore, at least 70 patients per center can be expected to qualify for assessment.

For ethical considerations, we think that the lack of evidence for the risk of hernia recurrence with biological meshes is balanced by the expected improvement of postoperative, chronic, inguinal pain and the advantage of avoiding a life-long implant in the patient. As both mesh types are CE-certified, already in frequent clinical use for various hernia repairs and the operation techniques do not vary from the standard procedures, the medical risk of hernia therapy in this trial is no different compared to routine clinical care.

As there are no multicenter, prospective studies yet researching the possible advantages of biological matrices in inguinal hernia repair and biological meshes are already frequently used, the need for a reasonably sized trial is apparent. The results of the BIOLAP trial will be published in peer-reviewed journals. A direct impact of the results on hernia repair standard procedure should be expected.

## Trial status

This trial protocol is published using version 3 on 16 October 2017 as approved by the Ethical Committee of the University of Witten/Herdecke. Recruitment started on 17 August 2017 and is estimated to be completed in January 2020.

## Additional file

Additional file 1: Standard Protocol Items: Recommendations for Interventional Trials (SPIRIT) 2013 Checklist: recommended items to address in a clinical trial protocol and related documents. (DOC 123 kb)

## Abbreviations

CE-certified: *Conformité Européenne*-certified; certified conformity with health, safety and environmental protection standards for products sold within the European Economic Area
CT scan: Computed tomography scan
DALY: Disability-adjusted life year
EHS: European Hernia Society
IEHS: International Endo Hernia Society
IIT: Investigator-initiated trial
ITT: Intention-to-treat
LOCF: Last observation carried forward
MRI scan: Magnetic resonance imaging scan
RCT: Randomized controlled trial
SD: Standard deviation
TAPP: Transabdominal preperitoneal patch plasty
TEP: Total extraperitoneal patch plasty
VAS: Visual analog scale

## References

[1] Fitzgibbons RJJ, Forse RA (2015). Groin hernias in adults. N Engl J Med.

[2] Primatesta P, Goldacre M (1996). Inguinal hernia repair: incidence of elective and emergency surgery, readmission and mortality. Int J Epidemiol.

[3] Bay-Nielsen M (2001). Quality assessment of 26 304 herniorrhaphies in Denmark: a prospective nationwide study. Lancet.

[4] Murray CJL (2012). Disability-adjusted life years (DALYs) for 291 diseases and injuries in 21 regions, 1990–2010: a systematic analysis for the Global Burden of Disease Study 2010. Lancet.

[5] Jacob D (2015). Perioperative outcome of unilateral versus bilateral inguinal hernia repairs in TAPP technique: analysis of 15,176 cases from the Herniamed Registry. Surg Endosc.

[6] HerniaSurge Group (2018). International guidelines for groin hernia management. Hernia.

[7] Macintyre IMC (2003). Best practice in groin hernia repair. Br J Surg.

[8] Pfeffer F (2008). Operation der beidseitigen Leistenhernie—Sequenziell oder simultan? [Repair of bilateral inguinal hernias—sequential or simultaneous?]. Zentralblatt Chirurgie.

[9] Wauschkuhn C, et al. Laparoscopic inguinal hernia repair: gold standard in bilateral hernia repair? Results of more than 2800 patients in comparison to literature. 2010;24:3026–30. 10.1007/s00464-010-1079-x. 10.1007/s00464-010-1079-x 20454807

[10] Berger D (2016). Evidence-based hernia treatment in adults. Deutsches Arzteblatt Int.

[11] Simons MP (2009). European Hernia Society guidelines on the treatment of inguinal hernia in adult patients. Hernia.

[12] Scott N (2002). Open mesh versus non-mesh for repair of femoral and inguinal hernia.

[13] EU Hernia Trialists Collaboration (2002). Repair of groin hernia with synthetic mesh: meta-analysis of randomized controlled trials. Ann Surg.

[14] Bochicchio GV (2014). Biologic vs synthetic inguinal hernia repair: 1-year results of a randomized double-blinded trial. J Am College Surg.

[15] Agresta F, Bedin N (2008). Transabdominal laparoscopic inguinal hernia repair: is there a place for biological mesh?. Hernia.

[16] Kockerling F (2015). Biological meshes for inguinal hernia repair – Review of the Literature. Front Surg.

[17] Fang Z (2015). Biologic mesh versus synthetic mesh in open inguinal hernia repair: system review and meta-analysis. ANZ J Surg.

[18] Sauerland S (2003). Fingers, hands or patients? The concept of independent observations. J Hand Surg.

[19] Bittner R (2011). Guidelines for laparoscopic (TAPP) and endoscopic (TEP) treatment of inguinal Hernia [International Endohernia Society (IEHS)]. Surg Endosc.

[20] Miserez M (2007). The European Hernia Society groin hernia classication: simple and easy to remember. Hernia.

[21] Köckerling F (2015). How long do we need to follow-up our hernia patients to find the real recurrence rate?. Front Surg.

